# 1-[(2*R*,4a*R*,8*R*,8a*R*)-8-Hy­droxy-4a,8-di­methyl­perhydronaphthalen-2-yl]ethan-1-one

**DOI:** 10.1107/S1600536811004454

**Published:** 2011-02-12

**Authors:** Mohamed Tebbaa, Ahmed Benharref, Moha Berraho, Jean-Claude Daran, Mohamed Akssira, Ahmed Elhakmaoui

**Affiliations:** a Laboratoire de Chimie Bioorganique et Analytique, URAC 22, BP 146, FSTM, Université Hassan II, Mohammedia–Casablanca 20810 Mohammedia, Morocco; b Laboratoire de Chimie Biomoleculaire, Substances Naturelles et Réactivité, URAC16, Université Cadi Ayyad, Faculté des Sciences Semlalia, BP 2390, Bd My Abdellah, 40000 Marrakech, Morocco; c Laboratoire de Chimie de Coordination, 205 route de Narbonne, 31077 Toulouse Cedex 04, France

## Abstract

The title compound, C_14_H_24_O_2_, was synthesized from ilicic acid, which was isolated from the aerial part of *Inula Viscosa­* (L) Aiton [or *Dittrichia Viscosa­* (L) Greuter]. The mol­ecule contains two fused six-membered rings, which both display a chair conformation. In the crystal, mol­ecules are linked into chains propagating along the *b* axis by inter­molecular O—H⋯O hydrogen bonds.

## Related literature

For the synthesis, see: Barrero *et al.* (2009 ▶). For the medicinal inter­est in *Inula Viscosa­* (L) Aiton [or *Dittrichia Viscosa­* (L) Greuter], see: Shtacher & Kasshman, (1970 ▶); Bohlmann *et al.* (1977 ▶); Chiappini *et al.* (1982 ▶) and for the pharmacological inter­est, see: Azoulay *et al.* (1986 ▶); Bohlmann *et al.* (1977 ▶); Ceccherelli *et al.* (1988 ▶). For background to phytochemical studies of plants, see: Geissman & Toribio (1967 ▶). For conformational analysis, see: Cremer & Pople (1975 ▶). 
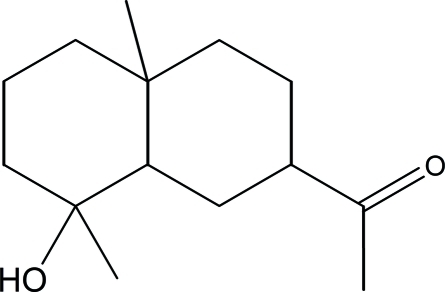


## Experimental

### 

#### Crystal data

C_14_H_24_O_2_
*M* *_r_* = 224.33
Monoclinic, 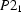

*a* = 6.4919 (7) Å
*b* = 9.4057 (9) Å
*c* = 10.3638 (11) Å
β = 97.286 (10)°
*V* = 627.71 (11) Å^3^
*Z* = 2
Mo *K*α radiation
μ = 0.08 mm^−1^
*T* = 180 K
0.6 × 0.25 × 0.15 mm

#### Data collection

Agilent Eos Gemini Ultra diffractometer
6571 measured reflections
1362 independent reflections
1262 reflections with *I* > 2σ(*I*)
*R*_int_ = 0.047

#### Refinement

*R*[*F* ^2^ > 2σ(*F* ^2^)] = 0.043
*wR*(*F* ^2^) = 0.119
*S* = 1.09
1362 reflections
152 parameters
1 restraint
H atoms treated by a mixture of independent and constrained refinement
Δρ_max_ = 0.28 e Å^−3^
Δρ_min_ = −0.24 e Å^−3^

### 

Data collection: *CrysAlis PRO* (Agilent, 2010 ▶); cell refinement: *CrysAlis PRO*; data reduction: *CrysAlis PRO*; program(s) used to solve structure: *SHELXL97* (Sheldrick, 2008 ▶); program(s) used to refine structure: *SHELXL97* (Sheldrick, 2008 ▶); molecular graphics: *ORTEP-3 for Windows* (Farrugia, 1997 ▶) and *PLATON* (Spek, 2009 ▶); software used to prepare material for publication: *WinGX* (Farrugia, 1999 ▶).

## Supplementary Material

Crystal structure: contains datablocks I, global. DOI: 10.1107/S1600536811004454/fj2393sup1.cif

Structure factors: contains datablocks I. DOI: 10.1107/S1600536811004454/fj2393Isup2.hkl

Additional supplementary materials:  crystallographic information; 3D view; checkCIF report

## Figures and Tables

**Table 1:** Hydrogen-bond geometry (Å, °)

*D*—H⋯*A*	*D*—H	H⋯*A*	*D*⋯*A*	*D*—H⋯*A*
O1—H2⋯O2^i^	0.84 (3)	2.05 (3)	2.883 (2)	169 (3)

Symmetry code: (i) 
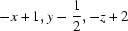
.

**Fig. 1.**
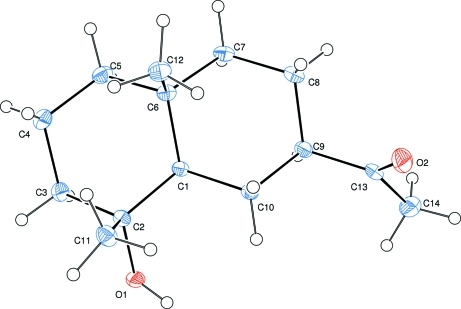
Molecular structure of the title compound with the atom-labelling scheme. Displacement ellipsoids are drawn at the 30% probability level. H atoms are represented as small spheres of arbitrary radii.

**Fig. 2.**
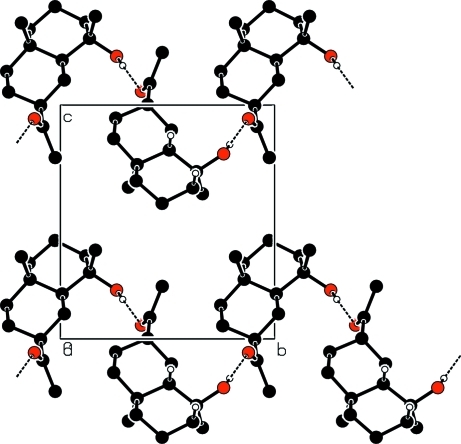
Partial packing view showing the O—H···O interactions (dashed lines)and the formation of a chain parallel to the b axis. H atoms not involved in hydrogen bonding have been omitted for clarity. [Symmetry code: (i)1 - x,-1/2 + y,-z + 2]

## supplementary crystallographic information

### Comment

The ilicic acid is one of the main components of the extracts of the aerial parts Inula viscose. This natural acid is a major constituent of the dichloromethane extract of the Inula Viscosa (*L*) Aiton [or Dittrichia Viscosa (*L*) Greuter]. This plant is widespread in Mediterranean area and extends to the Atlantic cost of Morocco. It is a well known medicinal plant (Shtacher & Kasshman, 1970; Chiappini *et al.*, 1982) and has some pharmacological activities (Azoulay *et al.*, 1986). the Inula Viscosa (*L*) Aiton has been the subject of chemical investigation in terms of isolating sesquiterpene lactones (Bohlmann *et al.*, 1977), sesquiterpene acids (Ceccherelli *et al.*, 1988; Geissman *et al.*1967). The literature report one article on the transformation of the ilicic acid (Barrero *et al.*, 2009). In order to prepare products with high added value, used in the industry pharmacological or cosmetic, we have studied the reactivity of this acid. Thus, with the reaction Curtius, we synthesized the title compound(1*R*, 2*R*, 6*R*, 9*R*)-9-acethyl-2,6-dimethylbicyclo [4.4.0]decan-2-ol) with à yield 50%. The structure of this new derivative of ilicic acid was determined by NMR spectral analysis of 1H, 13 C and mass spectroscopy and confirmed by its single-crystal X-ray structure. The molecule is built up from two fused six-membered rings. The molecular structure of (I),Fig.1, shows the two rings to adopt a perfect chair conformation as indicated by Cremer & Pople (1975) puckering parameters Q(T)= 0.554 (2)Å and spherical polar angle θ = 178.1 (2)° with φ = 36 (7)° for the first ring (C1,C2··· C6) and Q(T)= 0.597 (2)Å with a spherical polar angle θ = 178.71 (19)° and φ = 108 (5)° for the second ring (C1, C6···C10)(Cremer and Pople,1975). In the crystal structure, molecules are linked into chains (Fig. 2) running along the *b* axis by intermolecular O—H···O hydrogen bonds (Table 1) involving the O1 and O2 atoms.

### Experimental

A solution containing the ilicic acid 1 g (3.96 mmol) and Et3N 0.82 ml (5.895 mmol) in dry THF (100 ml) was cooled at -10 °C. Ethyl chloroformate 0,56 ml (5.95 mm l) was added dropwise and the reaction mixture was stirred at this temperature for 1 h. A solution of NaN3 0.43 g (6.74 mmol) in H2O (10 ml) was then added in one portion. After 1.5 h at 0 °C, the resulting heterogeous mixture was filttered, the organic solvent was removed under reduced pressure and the aqueous phase was extracted tree time with ether (3 × 50 ml). The combined organic layers were dried over MgSO4, and concentrated *in vacuo*. The crude acyl-azide was then dissolved in toluene (50 ml) and the resulting solution was refluxed for 1 h. Then a solution of hydrochloric acid at 10% was added to the reaction mixture which is remized at reflux for 2 h. After extraction, the organic phase is washed with water and brine, dried over anhydrous Na2SO4, filtered and concentrated under vacuum. The product was purified by column chromatography over silica gel (hexane/ethyl acetate 95/5). The title compound was recrystallized in dichloromethane.

### Refinement

Except H2, all H atoms were fixed geometrically and treated as riding with C—H = 0.96 Å (methyl),0.97 Å (methylene), 0.98Å (methine) with *U*_iso_(H) = 1.2Ueq(methylene, methine and OH) or *U*_iso_(H) = 1.5Ueq(methyl). In the absence of significant anomalous scattering, the absolute configuration could not be reliably determined and thus 1167 Friedel pairs were merged and any references to the Flack parameter were removed.

### Crystal data

**Table:**

C_14_H_24_O_2_	*F*(000) = 248
*M**_r_* = 224.33	*D*_x_ = 1.187 Mg m^−^^3^
Monoclinic, *P*2_1_	Mo *K*α radiation, λ = 0.71073 Å
Hall symbol: P 2yb	Cell parameters from 6571 reflections
*a* = 6.4919 (7) Å	θ = 2.9–26.4°
*b* = 9.4057 (9) Å	µ = 0.08 mm^−^^1^
*c* = 10.3638 (11) Å	*T* = 180 K
β = 97.286 (10)°	Prism, colourless
*V* = 627.71 (11) Å^3^	0.6 × 0.25 × 0.15 mm
*Z* = 2	

### Data collection

**Table:**

Agilent Eos Gemini Ultra diffractometer	1262 reflections with *I* > 2σ(*I*)
Radiation source: fine-focus sealed tube	*R*_int_ = 0.047
graphite	θ_max_ = 26.4°, θ_min_ = 2.9°
Detector resolution: 16.1978 pixels mm^-1^	*h* = −8→8
φ and ω scans	*k* = −11→11
6571 measured reflections	*l* = −12→12
1362 independent reflections	

### Refinement

**Table:**

Refinement on *F*^2^	Primary atom site location: structure-invariant direct methods
Least-squares matrix: full	Secondary atom site location: difference Fourier map
*R*[*F*^2^ > 2σ(*F*^2^)] = 0.043	Hydrogen site location: inferred from neighbouring sites
*wR*(*F*^2^) = 0.119	H atoms treated by a mixture of independent and constrained refinement
*S* = 1.09	*w* = 1/[σ^2^(*F*_o_^2^) + (0.0935*P*)^2^ + 0.0033*P*] where *P* = (*F*_o_^2^ + 2*F*_c_^2^)/3
1362 reflections	(Δ/σ)_max_ < 0.001
152 parameters	Δρ_max_ = 0.28 e Å^−^^3^
1 restraint	Δρ_min_ = −0.24 e Å^−^^3^

### Special details

**Table:**

Geometry. All s.u.'s (except the s.u. in the dihedral angle between two l.s. planes) are estimated using the full covariance matrix. The cell s.u.'s are taken into account individually in the estimation of s.u.'s in distances, angles and torsion angles; correlations between s.u.'s in cell parameters are only used when they are defined by crystal symmetry. An approximate (isotropic) treatment of cell s.u.'s is used for estimating s.u.'s involving l.s. planes.
Refinement. Refinement of *F*^2^ against ALL reflections. The weighted *R*-factor *wR* and goodness of fit *S* are based on *F*^2^, conventional *R*-factors *R* are based on *F*, with *F* set to zero for negative *F*^2^. The threshold expression of *F*^2^ > 2σ(*F*^2^) is used only for calculating *R*-factors(gt) *etc*. and is not relevant to the choice of reflections for refinement. *R*-factors based on *F*^2^ are statistically about twice as large as those based on *F*, and *R*- factors based on ALL data will be even larger.

### Fractional atomic coordinates and isotropic or equivalent isotropic displacement parameters (Å^2^)

**Table:**

	*x*	*y*	*z*	*U*_iso_*/*U*_eq_	
H2	0.822 (5)	0.709 (4)	0.829 (3)	0.039 (8)*	
C1	0.8653 (3)	0.9919 (2)	0.80775 (19)	0.0185 (4)	
H1	0.9924	0.9851	0.8695	0.022*	
C2	0.8777 (3)	0.8642 (2)	0.7154 (2)	0.0214 (4)	
C3	1.0704 (3)	0.8816 (2)	0.6454 (2)	0.0261 (5)	
H3A	1.1934	0.8693	0.7081	0.031*	
H3B	1.0705	0.8072	0.5806	0.031*	
C4	1.0826 (3)	1.0251 (3)	0.5791 (2)	0.0296 (5)	
H4A	0.9684	1.0339	0.5095	0.036*	
H4B	1.2114	1.0311	0.5411	0.036*	
C5	1.0729 (3)	1.1462 (2)	0.6757 (2)	0.0269 (5)	
H5A	1.0766	1.2360	0.6300	0.032*	
H5B	1.1947	1.1420	0.7403	0.032*	
C6	0.8776 (3)	1.1420 (2)	0.7452 (2)	0.0220 (5)	
C7	0.9014 (4)	1.2512 (2)	0.8558 (2)	0.0262 (5)	
H7A	1.0348	1.2372	0.9079	0.031*	
H7B	0.9008	1.3459	0.8186	0.031*	
C8	0.7305 (4)	1.2420 (2)	0.9442 (2)	0.0267 (5)	
H8A	0.5973	1.2638	0.8945	0.032*	
H8B	0.7565	1.3112	1.0137	0.032*	
C9	0.7245 (3)	1.0926 (2)	1.0020 (2)	0.0225 (4)	
H9	0.8596	1.0737	1.0527	0.027*	
C10	0.6895 (3)	0.9817 (2)	0.89272 (19)	0.0208 (4)	
H10A	0.6867	0.8871	0.9298	0.025*	
H10B	0.5572	0.9987	0.8402	0.025*	
C11	0.6816 (3)	0.8367 (3)	0.6205 (2)	0.0281 (5)	
H11A	0.5633	0.8342	0.6674	0.042*	
H11B	0.6942	0.7472	0.5776	0.042*	
H11C	0.6643	0.9114	0.5570	0.042*	
C12	0.6859 (4)	1.1824 (3)	0.6488 (2)	0.0291 (5)	
H12A	0.6890	1.1314	0.5688	0.044*	
H12B	0.6873	1.2827	0.6321	0.044*	
H12C	0.5619	1.1581	0.6854	0.044*	
C13	0.5607 (4)	1.0824 (2)	1.0919 (2)	0.0254 (5)	
C14	0.6262 (4)	1.0246 (3)	1.2248 (2)	0.0365 (6)	
H14A	0.5094	1.0239	1.2732	0.055*	
H14B	0.7341	1.0832	1.2688	0.055*	
H14C	0.6770	0.9293	1.2181	0.055*	
O1	0.9229 (2)	0.73764 (16)	0.79125 (15)	0.0258 (4)	
O2	0.3823 (3)	1.1216 (2)	1.05879 (17)	0.0368 (4)	

### Atomic displacement parameters (Å^2^)

**Table:**

	*U*^11^	*U*^22^	*U*^33^	*U*^12^	*U*^13^	*U*^23^
C1	0.0181 (9)	0.0150 (9)	0.0221 (10)	0.0001 (7)	0.0013 (7)	0.0000 (7)
C2	0.0234 (11)	0.0173 (9)	0.0236 (10)	0.0027 (8)	0.0035 (8)	0.0001 (8)
C3	0.0265 (11)	0.0247 (11)	0.0283 (11)	0.0026 (8)	0.0079 (8)	−0.0005 (9)
C4	0.0300 (11)	0.0311 (12)	0.0299 (11)	−0.0018 (9)	0.0119 (9)	0.0033 (9)
C5	0.0256 (11)	0.0237 (11)	0.0322 (11)	−0.0027 (8)	0.0065 (8)	0.0053 (9)
C6	0.0200 (10)	0.0188 (10)	0.0269 (10)	−0.0005 (7)	0.0017 (7)	0.0025 (8)
C7	0.0299 (11)	0.0163 (10)	0.0319 (11)	−0.0033 (8)	0.0016 (9)	−0.0003 (8)
C8	0.0308 (11)	0.0180 (10)	0.0309 (11)	0.0017 (9)	0.0024 (9)	−0.0025 (8)
C9	0.0258 (10)	0.0185 (10)	0.0229 (10)	0.0003 (8)	0.0026 (8)	−0.0020 (7)
C10	0.0236 (10)	0.0149 (9)	0.0241 (10)	0.0001 (8)	0.0040 (7)	−0.0014 (7)
C11	0.0253 (11)	0.0266 (11)	0.0321 (11)	−0.0011 (8)	0.0024 (9)	−0.0068 (9)
C12	0.0298 (12)	0.0263 (11)	0.0304 (11)	0.0037 (9)	0.0003 (9)	0.0055 (9)
C13	0.0325 (12)	0.0149 (9)	0.0296 (11)	0.0006 (8)	0.0070 (8)	−0.0058 (8)
C14	0.0463 (14)	0.0321 (13)	0.0324 (12)	−0.0030 (11)	0.0097 (10)	0.0025 (10)
O1	0.0279 (8)	0.0172 (7)	0.0341 (8)	0.0036 (6)	0.0103 (6)	0.0028 (6)
O2	0.0336 (9)	0.0360 (10)	0.0429 (9)	0.0095 (7)	0.0132 (7)	0.0005 (8)

### Geometric parameters (Å, °)

**Table:**

C1—C10	1.530 (3)	C8—C9	1.530 (3)
C1—C2	1.545 (3)	C8—H8A	0.9700
C1—C6	1.560 (3)	C8—H8B	0.9700
C1—H1	0.9800	C9—C13	1.503 (3)
C2—O1	1.436 (3)	C9—C10	1.535 (3)
C2—C11	1.529 (3)	C9—H9	0.9800
C2—C3	1.532 (3)	C10—H10A	0.9700
C3—C4	1.521 (3)	C10—H10B	0.9700
C3—H3A	0.9700	C11—H11A	0.9600
C3—H3B	0.9700	C11—H11B	0.9600
C4—C5	1.524 (3)	C11—H11C	0.9600
C4—H4A	0.9700	C12—H12A	0.9600
C4—H4B	0.9700	C12—H12B	0.9600
C5—C6	1.536 (3)	C12—H12C	0.9600
C5—H5A	0.9700	C13—O2	1.222 (3)
C5—H5B	0.9700	C13—C14	1.493 (3)
C6—C7	1.533 (3)	C14—H14A	0.9600
C6—C12	1.541 (3)	C14—H14B	0.9600
C7—C8	1.528 (3)	C14—H14C	0.9600
C7—H7A	0.9700	O1—H2	0.85 (3)
C7—H7B	0.9700		
			
C10—C1—C2	114.14 (16)	H7A—C7—H7B	107.7
C10—C1—C6	112.18 (15)	C7—C8—C9	110.07 (17)
C2—C1—C6	115.90 (15)	C7—C8—H8A	109.6
C10—C1—H1	104.3	C9—C8—H8A	109.6
C2—C1—H1	104.3	C7—C8—H8B	109.6
C6—C1—H1	104.3	C9—C8—H8B	109.6
O1—C2—C11	107.93 (18)	H8A—C8—H8B	108.2
O1—C2—C3	103.02 (16)	C13—C9—C8	110.87 (17)
C11—C2—C3	112.14 (17)	C13—C9—C10	111.32 (17)
O1—C2—C1	109.18 (15)	C8—C9—C10	110.12 (16)
C11—C2—C1	115.19 (16)	C13—C9—H9	108.1
C3—C2—C1	108.64 (17)	C8—C9—H9	108.1
C4—C3—C2	113.69 (17)	C10—C9—H9	108.1
C4—C3—H3A	108.8	C1—C10—C9	109.28 (15)
C2—C3—H3A	108.8	C1—C10—H10A	109.8
C4—C3—H3B	108.8	C9—C10—H10A	109.8
C2—C3—H3B	108.8	C1—C10—H10B	109.8
H3A—C3—H3B	107.7	C9—C10—H10B	109.8
C3—C4—C5	110.96 (17)	H10A—C10—H10B	108.3
C3—C4—H4A	109.4	C2—C11—H11A	109.5
C5—C4—H4A	109.4	C2—C11—H11B	109.5
C3—C4—H4B	109.4	H11A—C11—H11B	109.5
C5—C4—H4B	109.4	C2—C11—H11C	109.5
H4A—C4—H4B	108.0	H11A—C11—H11C	109.5
C4—C5—C6	113.21 (17)	H11B—C11—H11C	109.5
C4—C5—H5A	108.9	C6—C12—H12A	109.5
C6—C5—H5A	108.9	C6—C12—H12B	109.5
C4—C5—H5B	108.9	H12A—C12—H12B	109.5
C6—C5—H5B	108.9	C6—C12—H12C	109.5
H5A—C5—H5B	107.7	H12A—C12—H12C	109.5
C7—C6—C5	108.80 (16)	H12B—C12—H12C	109.5
C7—C6—C12	108.44 (18)	O2—C13—C14	121.2 (2)
C5—C6—C12	109.67 (17)	O2—C13—C9	121.8 (2)
C7—C6—C1	107.52 (17)	C14—C13—C9	117.03 (19)
C5—C6—C1	107.90 (16)	C13—C14—H14A	109.5
C12—C6—C1	114.38 (18)	C13—C14—H14B	109.5
C8—C7—C6	113.48 (17)	H14A—C14—H14B	109.5
C8—C7—H7A	108.9	C13—C14—H14C	109.5
C6—C7—H7A	108.9	H14A—C14—H14C	109.5
C8—C7—H7B	108.9	H14B—C14—H14C	109.5
C6—C7—H7B	108.9	C2—O1—H2	113 (2)

### Hydrogen-bond geometry (Å, °)

**Table:**

*D*—H···*A*	*D*—H	H···*A*	*D*···*A*	*D*—H···*A*
O1—H2···O2^i^	0.84 (3)	2.05 (3)	2.883 (2)	169 (3)

Symmetry codes: (i) −*x*+1, *y*−1/2, −*z*+2.
